# Hypnosis measured with monitors of anesthetic depth – EEG changes during the test for Harvard Group Scale of Hypnotic Susceptibility

**DOI:** 10.3389/fpsyg.2023.1267658

**Published:** 2023-12-19

**Authors:** Nina Zech, Milena Seemann, Ernil Hansen

**Affiliations:** ^1^Department of Anesthesiology, University Hospital Regensburg, Regensburg, Germany; ^2^Department of Anaesthesiology, Agaplesion Diakonieklinikum Hamburg, Hamburg, Germany

**Keywords:** hypnotic susceptibility, suggestibility, bispectral index, cerebral state index, trance

## Abstract

**Introduction:**

Hypnotic trance can be defined as a non-ordinary state of consciousness that is accompanied by a number of neurophysiological changes, including brain electrophysiology. In addition to subjective measures, corresponding objective parameters are needed in experimental and clinical hypnosis research but are complex, impractical, or unspecific. A similar challenge exists for the measurement and monitoring of drug-induced hypnosis, namely general anesthesia. The observation of changes in EEG induced by narcotics has led to the development of monitors for the depth of anesthesia based on EEG parameters. We investigated whether two such monitors react to the induction and maintenance of hypnosis during a highly standardized procedure.

**Methods:**

A total of 56 volunteers were monitored for the bispectral index (BIS) and cerebral state index (CSI) (range 0–100, >95 considered “awake”) during the Harvard Group Scale of Hypnotic Susceptibility test. For this test, trance is induced by a taped text and followed by 12 tasks performed under hypnosis. In contrast to random forms of hypnosis, this represents a standardized, worldwide-established condition. According to the resulting score, participants were classified into suggestibility groups in order to evaluate whether the electrophysiological measurements of BIS and CIS indices differ between high and low suggestible persons. Furthermore, participants were asked to rate their hypnotic depth (HD, 1–10) at every task of the test.

**Results:**

Scores dropped significantly from a mean of 97.7 to 86.4 for BIS and from 94.6 to 77.7 for CSI with the induction of hypnosis to stay throughout hypnosis at levels of approximately 88.6 or 82.9, respectively. Results did not differ between high- and low-suggestible participants. The means of the subjective score of hypnotic depth and of the electrophysiological measurements showed a similar course. However, no correlation was found between BIS or CSI values and scores of hypnotic depths.

**Conclusion:**

Monitors for depth of anesthesia respond to changes in consciousness, including trance states of hypnosis. However, specificity is unclear. Practically, in hypnosis research with the exclusion of drug effects or sleep, these monitors might be helpful to test and compare the efficacy of induction texts and to detect disturbances of trance state.

## 1 Introduction

Hypnotic trance is a non-ordinary state of consciousness induced and utilized in hypnotherapy to present suggestions to a patient that elicit profound psychological and physiological effects (De Benedittis, 2015; Fernandez et al., 2022). The American Psychological Association defines hypnosis as “a state of consciousness involving focused attention and decreased peripheral awareness, characterized by an increase in the ability to respond to suggestions” (Elkins et al., 2015). Hypnosis can be characterized by functional changes in brain activity, as demonstrated by various neuroimaging techniques and electrophysiological measurements (Wolf et al., 2022). Although several articles describe parameters and claim that they could distinguish hypnosis from other states of consciousness such as relaxation or meditation, none has yet been validated to exclusively be characteristic of hypnosis. In addition, to allow for objective rather than merely subjective measures in experimental and clinical applications, less sophisticated and more feasible methods would be needed to monitor hypnotic trance and trance depth. A potential solution could be monitors that have been developed to evaluate another alteration of consciousness, namely the depth of general anesthesia. Several devices have been designed and extensively evaluated to derive scores from processed EEG to measure a patient's level of consciousness during general anesthesia (Roche and Mahon, 2021). Regardless of the company-specific algorithm that unfortunately is kept secret, these indices range from zero to 100 (“awake”) with a range of 40–60 aimed for a sufficient anesthetic depth. The widest distribution has been found in the bispectral index (BIS), especially with the intention of protecting patients from “intraoperative awareness” and its medical and legal consequences (Stein and Glick, 2016). The cerebral state index (CSI) is similar to BIS but far less common in application. A connection between hypnosis and narcosis in the monitoring of changes in consciousness is further supported by the fact that the terms “hypnosis” and “hypnotic depth” are used for both the induction of pharmacological and psychological hypnosis, which should not be confused and has to be considered in corresponding literature search. In addition, there is no strict specificity for narcosis in such monitoring. There exist discrepancies between these electrophysiological scores and clinical signs of anesthesia (Jensen et al., 2004). BIS responses have also been reported for sleep (Nieuwenhuijs et al., 2002) or acupressure-induced relaxation (Fassoulaki et al., 2003). Furthermore, changes in BIS have also been reported under various physiological conditions such as hypoglycemia, hypothermia, or muscle relaxation (Dahaba, 2005). Recently, there have been attempts to test non-pharmacological hypnosis with monitors of anesthetic depth, namely BIS (De Benedittis, 2008) or CSI (Bock, 2013; Haipt et al., 2017), all using unspecified trance induction texts published only in Italian or German.

We report on measurements of hypnotic trance using two indices derived from anesthesia monitors, namely the BIS and the CSI, applied simultaneously. Previously, in pilot studies, we evaluated different hypnotic induction techniques with these monitors (data not shown) and found that hypnotic brain responses vary with the specific technique and text of trance induction, which makes comparison difficult. Therefore, to make results comparable with others, we used for hypnosis induction and maintenance the Harvard Group Scale of Hypnotic Susceptibility (HGSHS:A; hereafter referred to only as HGSHS) test (Shor and Orne, 1962). This test begins with a standardized trance induction followed by 12 hypnotic phenomena and represents a standardized, worldwide, uniformly used form of hypnosis (Peter, 2023).

## 2 Materials and methods

### 2.1 Design and participants

After approval by the local ethics committee (EC University of Regensburg, vote 13-101-0040), an experimental study was performed with 56 volunteers after informed consent. The age of the participants was limited to 18–70 years. Exclusion criteria were also a severe systemic disease, i.e., a higher than II score on the American Society of Anesthesiologists (ASA) Physical Status Classification System, language barriers, or a pre-existing cognitive impairment. Special attention was paid to the exclusion of psychiatric disorders or the intake of psychiatric medication.

### 2.2 Simultaneous measurement of BIS and CSI

During the study trial, EEG-derived indices were continuously recorded by two monitors for depth of anesthesia, namely the Bispectral Index Scale monitor (BIS-monitor, VISTA^®^ bilateral monitoring system; Anandic Medical Systems, Switzerland) and the Cerebral State Monitor^®^ (CSM, cerebral state monitoring system; Danmeter, Denmark).

The BIS index is a numerically processed, clinically validated EEG parameter. Unlike traditional processed EEG parameters derived from spectral analysis, the BIS index is derived utilizing a composite of multiple advanced EEG signal processing techniques, including bispectral analysis, power spectral analysis, and time-domain analysis. The key EEG features identified from the database analysis include the degree of beta or high frequency (14–30 Hz) activation, the amount of low-frequency synchronization, the presence of nearly suppressed periods within the EEG, and the presence of isoelectric periods within the EEG (Sigl and Chamoun, 1994). The CSI algorithm is based on fuzzy logic and has four sub-parameters derived from time-domain analysis (burst ratio) and frequency-domain analysis (α-ratio, β-ratio, and β-ratio–α-ratio) of the EEG (Jensen et al., 2006; Cho et al., 2018).

**Figure 1:**
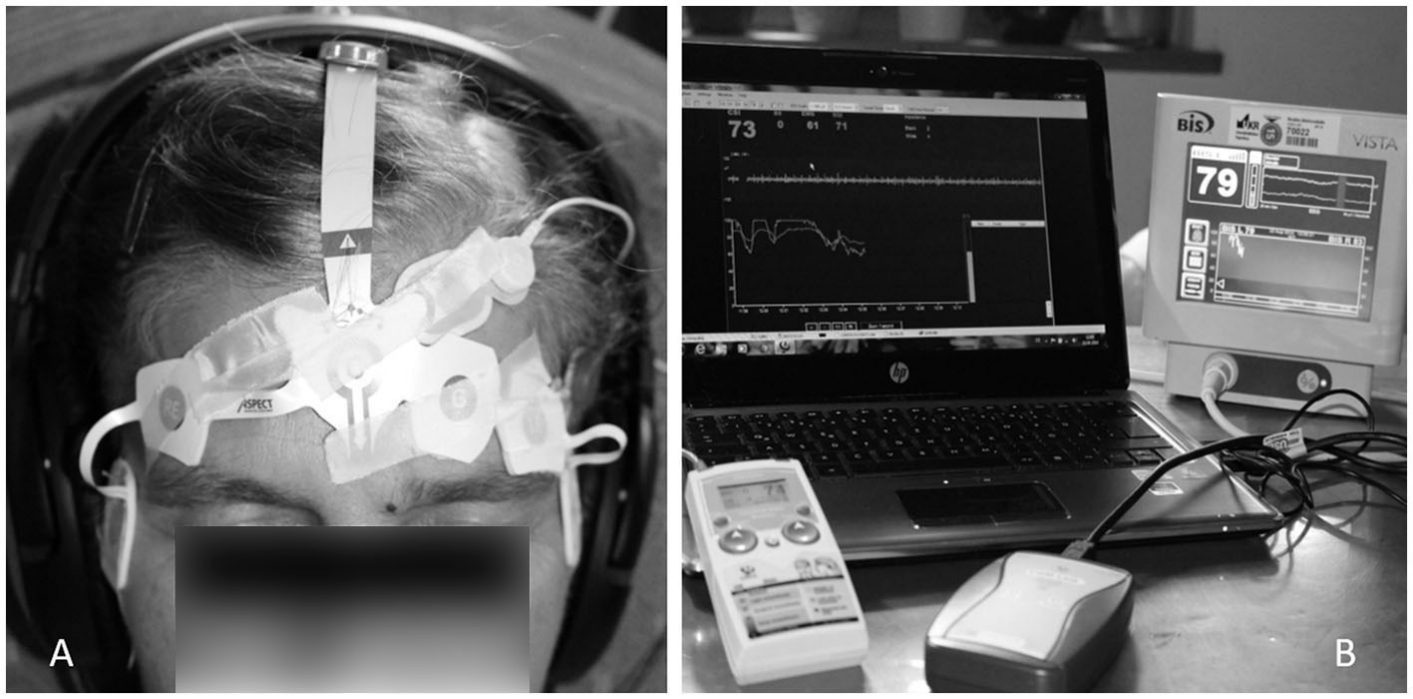
Participant with electrodes for bilateral BIS and CSI measurements placed on the forehead **(A)**. BIS VISTA^®^ bilateral monitoring system and Cerebral State Monitor^®^ for simultaneous recording of both index values **(B)**.

The measurement was conducted in a quiet room to avoid any disturbance. Participants were positioned slightly reclined on a comfortable chair with device-specific adhesive bilateral electrodes fixed on the forehead, following the manufacturer's instructions. The precise sensor positions are shown in Figure 1. The BIS sensor is a single-use component consisting of a plaster with (for bilateral registration 6) fixed electrodes. The sensor is placed with the central electrode at the center of the forehead, half a centimeter above the bridge of the nose, two electrodes above the left eyebrow, and an electrode midline between the edge of the eye and the hairline. The CSI electrodes were placed according to the operation manual. For high data quality, the skin was prepared with a skin preparation product (CSM Procedure Pack, Danmeter, Denmark), and the sensor was additionally fixed with adhesive tape. Data from both systems were immediately exported to a USB stick (BIS monitor) or wirelessly to the computer (CSM) using the CSM link software. Data for both indices (bispectral index scale = BIS and cerebral state index = CSI), recorded every second, were collected and stored in Excel Microsoft (Version 2010). Although BIS was registered bilaterally, only left-side signals were processed further to be comparable with unilateral BIS monitoring, where the left side is determined by the electrode assembly, as well as with CSI monitoring following the manufacturer‘s instructions.

### 2.3 Measurement of hypnosis during the Harvard Group Scale of Hypnotic Susceptibility test

After baseline measurements of both BIS and CSI (first “awake” value), every participant performed the test for HGSHS in the German version using the standardized audio file (Bongartz, 1985). The HGSHS test takes approximately 55 min. An introduction (7 min) is followed by a hypnotic induction (19 min) including two tasks (head falling and eye closure). At time point 2f, regardless of the individual time necessary to reach the trance state, induction is completed with the final request for eye closure in case the eyes did not close involuntarily before. This is followed by 10 more tasks (see Table 1). Finally, after two posthypnotic suggestions (ankle touching and amnesia), the trance is canceled by counting backward at the end of task 11. After the termination of the hypnotic trance, the 12th item of the test, i.e., recovery from amnesia, is verified. The efficacy of the suggestions, i.e., the quality with which each task was mastered, was evaluated by the subjects' self-assessment immediately after the termination of hypnosis. While participants filled out the test questionnaire, baseline values for both BIS and CSI were recorded again (second “awake” value). Participants were rated according to the scores as “low suggestible” (LS, scores 0–4), “medium suggestible” (MS, scores 5–8), and “high suggestible” (HS, scores 9–12) (Peter et al., 2015).

**Table 1:** Items of the test for the Harvard Group Scale of Hypnotic Susceptibility (HGSHS:A), their duration (min and sec), and the division into three phases.

**Tasks**	**Description**	**Duration**
1	Head drop (test suggestion)	3'30”
2 a-f	Eye closure	15'25”
3 a-b	Lowering left hand	5'05”
4	Immobility of right arm	2'55”
5	Finger lock	1'40”
6	Arm rigidity	2'25”
7	Attraction of palms	1'45”
8	Inhibition of head shaking	1'25”
9	Hallucination of a Fly	1'30”
10	Eye catalepsy	2'
11	Posthypnotic order (touching ankle)	3'50”
12	Reversal of posthypnotic amnesia	6'40”

### 2.4 Hypnotic depth

Together with the subjective evaluation of the performance in the various HGSHS tasks after the test, participants were requested to rate the hypnotic depth during each task on a scale of 1 to 10 (Pekala and Maurer, 2013).

### 2.5 Statistical analyses

For analyses, the 1^st^ min of the recording of BIS and CSM was discarded because of the latency time of both systems. For calculation, the recorded 1-sec-values were combined into periods of approximately 3 min. Accordingly, the long HGSHS item 2 was divided into 2a to 2f, and item 3 into 3a and 3b (see Table 1). Moreover, for comparisons, four phases were distinguished, and data were combined accordingly: “awake”, “introduction” (HGSHS item 1), “induction” (trance induction during HGSHS item 2), and “tasks” (during HGSHS items 1–11) (see Table 1). Variables were tested for normal distribution using the Kolmogorov–Smirnov–Lilliefors test. According to the detected normal distributions (*p* > 0.05), mixed factorial ANOVA was performed, including the four phases of HGSHS, the three groups of suggestibility (LS, MS, and HS), two age groups (18–30, 31–63, according to the median), and gender, followed by *post-hoc* Bonferroni-adjusted pair comparisons using Student's *t-*test. Means and SD were calculated for every item of the HGSHS test and used for data presentation. For direct comparison between BIS and HD, linear regression analysis for non-parametric data was performed using the results of HGSHS items 2 to 5 with the lowest levels of BIS, since a causal relationship could be expected. The two electrophysiological indices BIS and CSI were compared by linear correlation analysis for non-parametric data using the 17 periods during HGSHS testing (see **Figure 3**). Statistical analyses were performed with SPSS 24.0. *P*-values < 0.05 were considered to be statistically significant. The effect size was calculated at www.psychometrica.de.

## 3 Results

### 3.1 Baseline characteristics and hypnotic susceptibility scores

The age of participants varied between 18 and 63 years and showed two age peaks at 25 years (students) and 50 years (working adults). No dataset had to be discarded due to missing data. Participants‘ characteristics and baseline scores are shown in Table 2. Within the period of baseline recording, the BIS showed great robustness with an intraindividual variance of 0.1 ± 0.1 (mean of variance of 1-min-periods of index values in the awake state), in contrast to the CSI (intraindividual variance of 1.0 ± 0.6). In general, baseline BIS values were higher with less variation. Both BIS and CSI values showed a normal distribution in the various groups. Hypnotic suggestibility, according to the HGSHS-Score, was normally distributed without any relevant effect of sex or age. According to the usual grouping rules with HGSHS, 27% of participants were rated as “high suggestibles.”

**Table 2:** Baseline characteristics and score results of the study population (*n* = 56).

Age [Mean ± SD (median]	35 ± 14.5 (30.5)
Female sex [*n* (%)]	35 (63)
Baseline values (“awake”)^*^	
- BIS [Mean ± SD]	97.6 ± 0.2
- CSI [Mean ± SD]	94.6 ± 3.6
HGSHS-score (0–12) [Mean ± SD]	6.8 ± 2.7
Susceptibility groups [*n* (%)]	
- High suggestibles (9–12)	15 (27)
- Medium suggestibles (5–8)	31 (55)
- Low suggestibles (0–4)	10 (18)

HGSHS, Harvard Groupe Scale of Hypnotic Suggestibility; BIS, bispectral index scale; CSI, cerebral state index. ^*^Average of several awake phases.

### 3.2 BIS and CSI while performing the test for HGSHS

**Figure 2:**
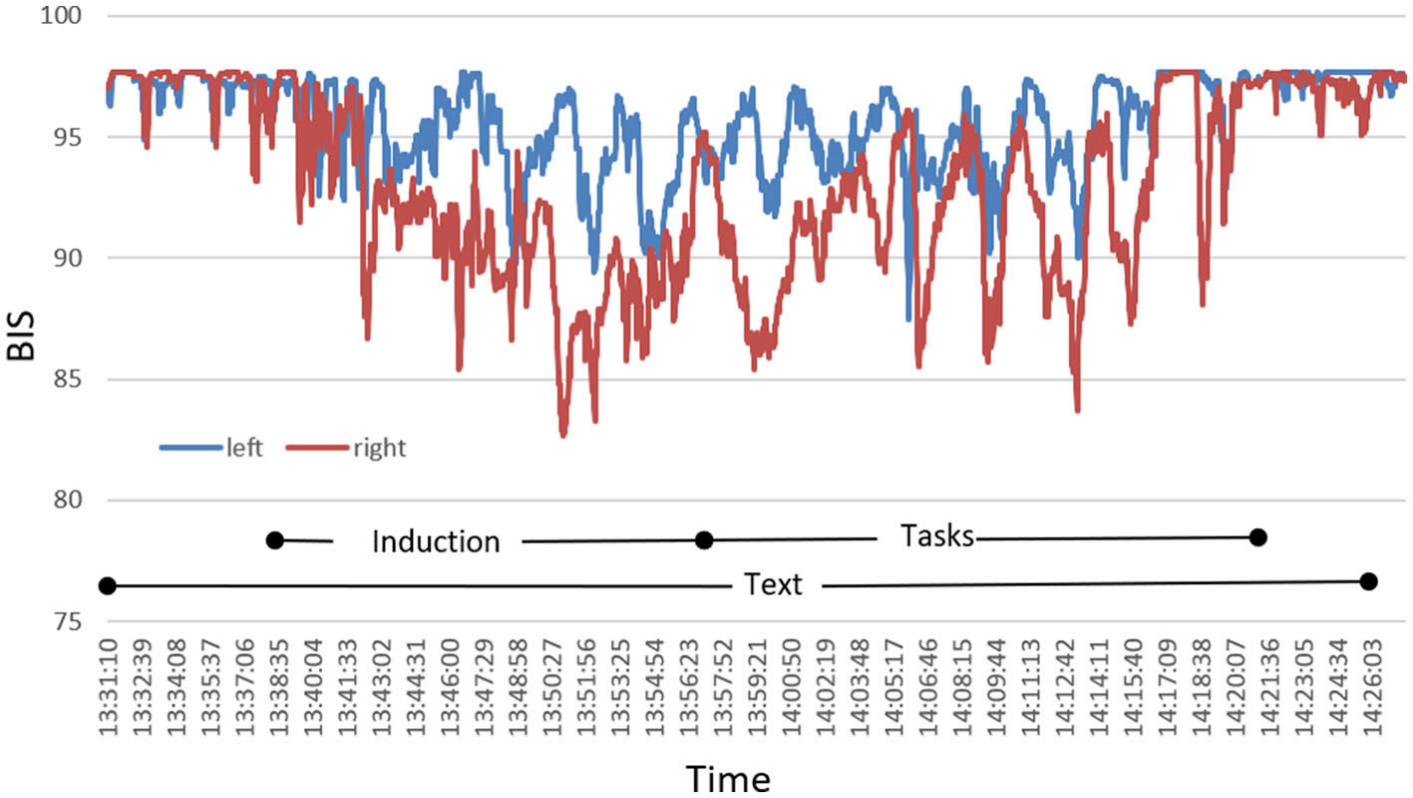
Exemplary presentation of a characteristic course of bilateral BIS during HGSHS testing of an individual participant with pronounced desynchronization. Index values recorded by the monitor continuously are plotted in intervals of 1 s.

During the HGSHS test, BIS was recorded bilaterally with electrodes placed both on the left and right forehead. As shown in Figure 2, in some participants, we observed marked desynchronization between left- and right-sided BIS. Since we did not find such differences consistently, we focused further analyses on the standard left-sided BIS in compliance with CSI, where left-side registration is recommended in the manufacturers' instructions.

**Figure 3:**
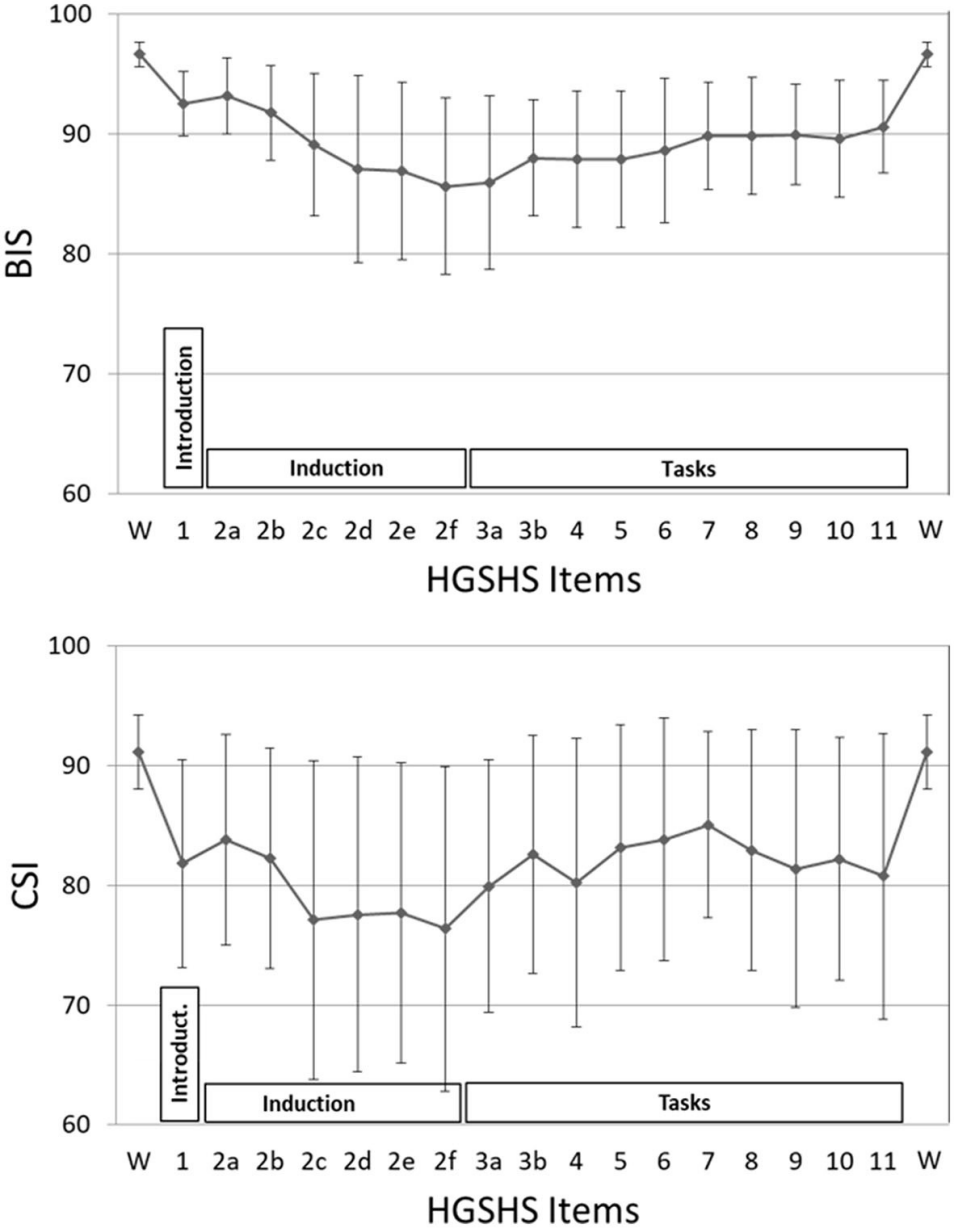
BIS and CSI values during the HGSHS test. Each point represents the mean of index values during a certain task (85–175 sec), or 3-min intervals during the introduction and induction phases, respectively. Phases: introduction (item 1), trance induction (item 2), hypnotic tasks (items 3–11) (see also Table 1). The mean values of all three phases were different from the awake baseline with statistical significance.

BIS and CSI were recorded simultaneously throughout the whole time of the HGSHS testing (Figure 3). Starting from the awake condition with a score of 97.7 (±1.0), the average BIS declined continuously, reaching its deepest mean score of 86.4 (±7.4) at the end of trance induction. Subsequently, during the hypnotic tasks, BIS raised slightly, reaching a plateau at approximately 90. After the termination of the hypnotic trance, values increased and reached the awake level again. Mixed ANOVA, used according to normal distributions of values, demonstrated differences only for the four phases of HGSHS testing, i.e., wake, introduction, trance induction, and the hypnotic tasks, not for gender, age, or suggestibility group (F = 72.6, *p* < 0.001). *Post-hoc* pairwise comparison using Student's *t-*test showed differences of statistical significance between the awake value and every other phase, with t = 12.2, 11.3, 16.0, and *p* < 0.001, respectively. In addition, the differences in the phases introduction and induction, as well as introduction and tasks, were significant with t = 6.5 and 10.0 (*p* < 0.001), respectively. There was no significant difference between the BIS values in the induction phase and task phase (t = 0.2, n.s.). Concerning the CSI values, we also found decreasing values during the induction from an average score of 94.6 (±3.6) to 77.2 (±14.4), but the decline showed more fluctuating values. The deepest point was also at the end of trance induction (2f in Figure 3). While performing the hypnotic tasks, the CSI level increased slightly and stayed below or at a level of 85, again with more fluctuation. After counting backward for the termination of the hypnotic trance, CSI increased to the initial awake level. The differences between the mean CSI of every HGSHS phase and the awake baseline were statistically significant, with t = 7.2, 8.3, 6.7, and *p* < 0.001, respectively. BIS responded to the induction phase and the task phase with an effect size (Cohen's d) of −2.1 and −3.1, respectively. For CSI, the corresponding effect sizes were −1.5 and −1.2, respectively.

### 3.3 Subjective trance depth (HD)

**Figure 4:**
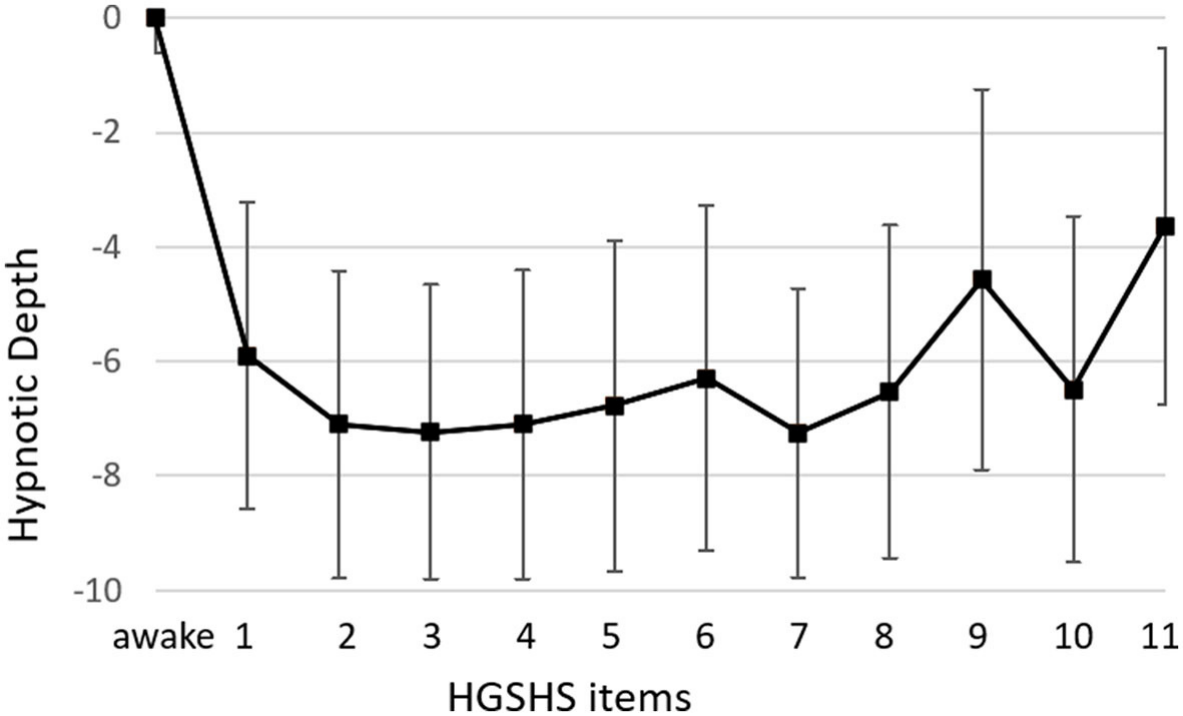
Course of hypnotic depth during HGSHS testing. Hypnotic depth was evaluated with a subjective score ranging from 1 to 10 after the test. Means and SD for every item of the HGSHS are shown.

**Figure 5:**
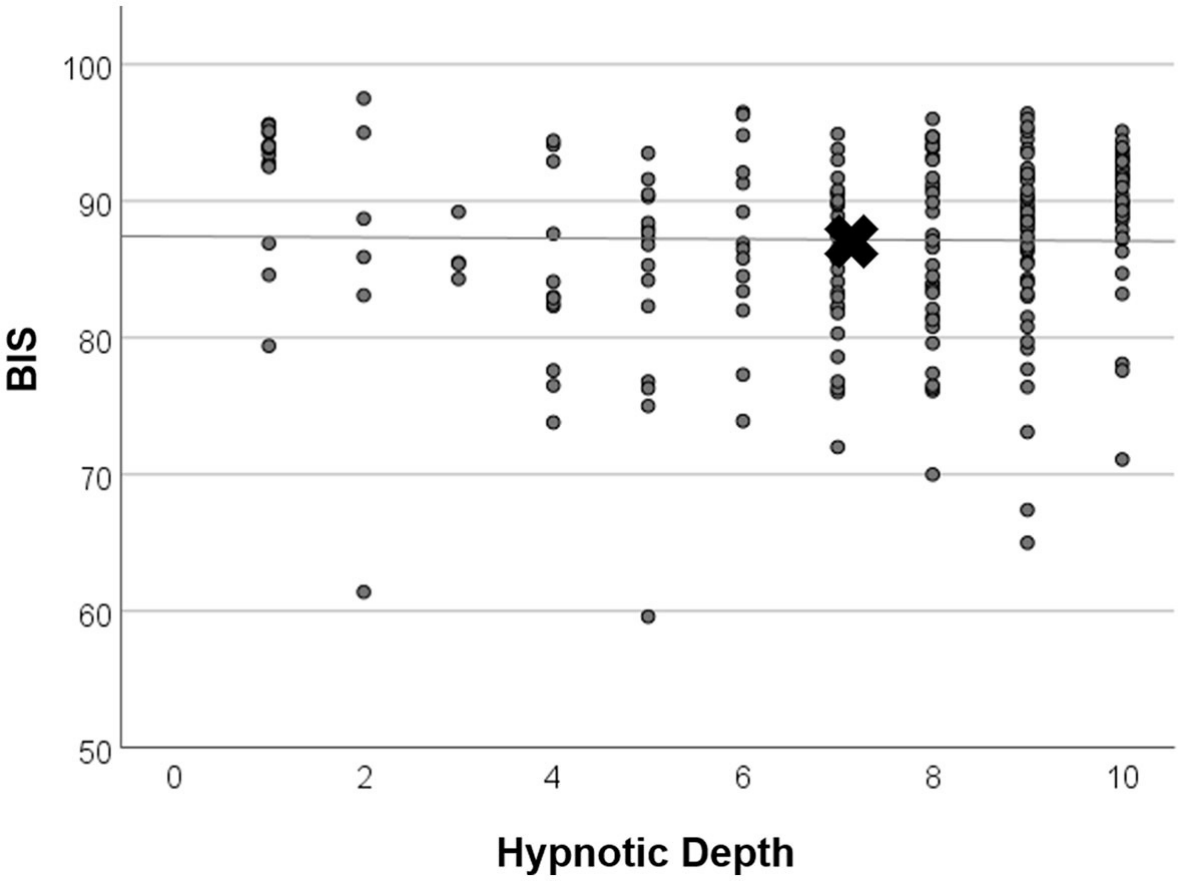
Linear regression analysis of BIS and hypnotic depth. Values were taken from time points items 2–5, where both mean BIS and mean HD showed low levels (see Figure 4). X marks the means of parameters. There was no significant relationship between BIS and HD (regression coefficient R^2^ < 0.001, n.s.).

The course of mean HD scores paralleled that of the electrophysiological indices (Figure 4). However, bivariate correlation analysis using values of HGSHS items 2–5, where both BIS and HD showed deep levels, revealed no significant interaction or causal relationship between the two parameters in the individuals, with a regression coefficient of R^2^ < 0.001, n.s. (Figure 5). In contrast, the subjective hypnotic depth score correlated with HGSHS (Spearman-Rho r = 0.74, *p* < 0.001).

### 3.4 Comparison between BIS and CSI values

**Figure 6:**
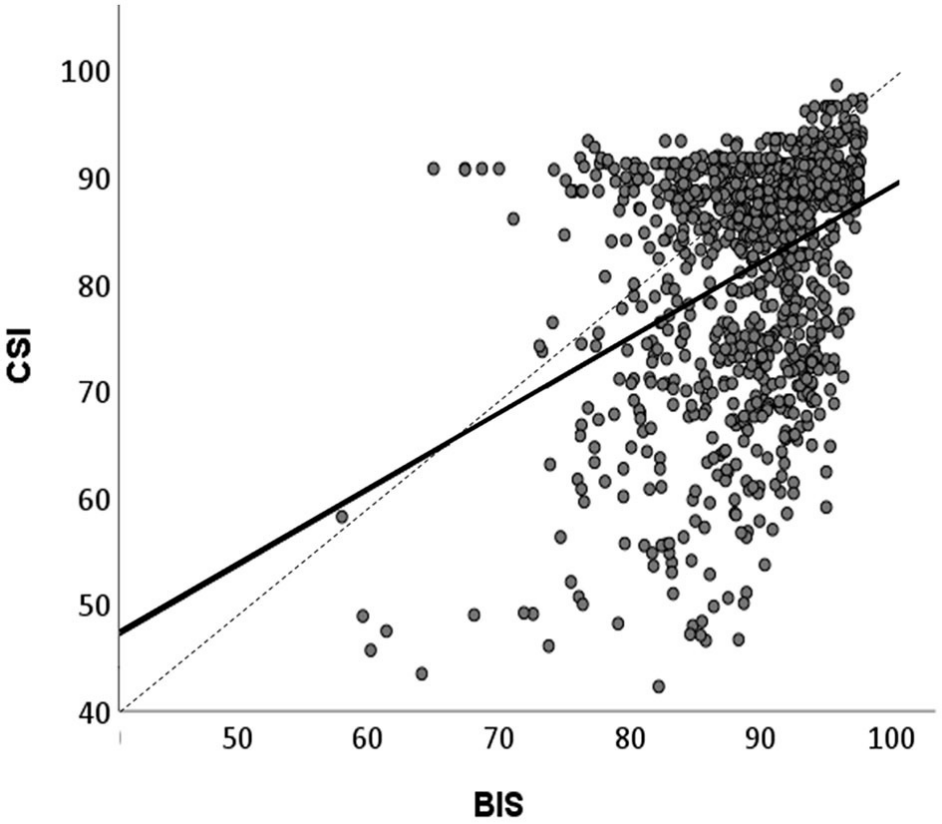
Linear correlation between BIS and CSI values recorded during HGSHS testing. Means of BIS and CSI values in the 17 time periods (approximately 3-min intervals, see Figure 3) were compared. Spearman‘s correlation coefficient r = 0.38, *p* < 0.001. CSI shows lower values than BIS.

The recording of BIS and CSI revealed comparable courses during HGSHS. Nevertheless, there were marked differences between the two devices. CSI already started at a deeper level than BIS, and during HGSHS, CSI consistently reached lower levels. In general, CSI values showed higher standard deviations, both for baseline and while performing the HGSHS test. Correlation analysis showed a significant linear relation between BIS and CSI values, with a Spearman-Rho correlation coefficient r = 0.37, *p* < 0.001 (Figure 6).

### 3.5 Confounding factors

**Figure 7:**
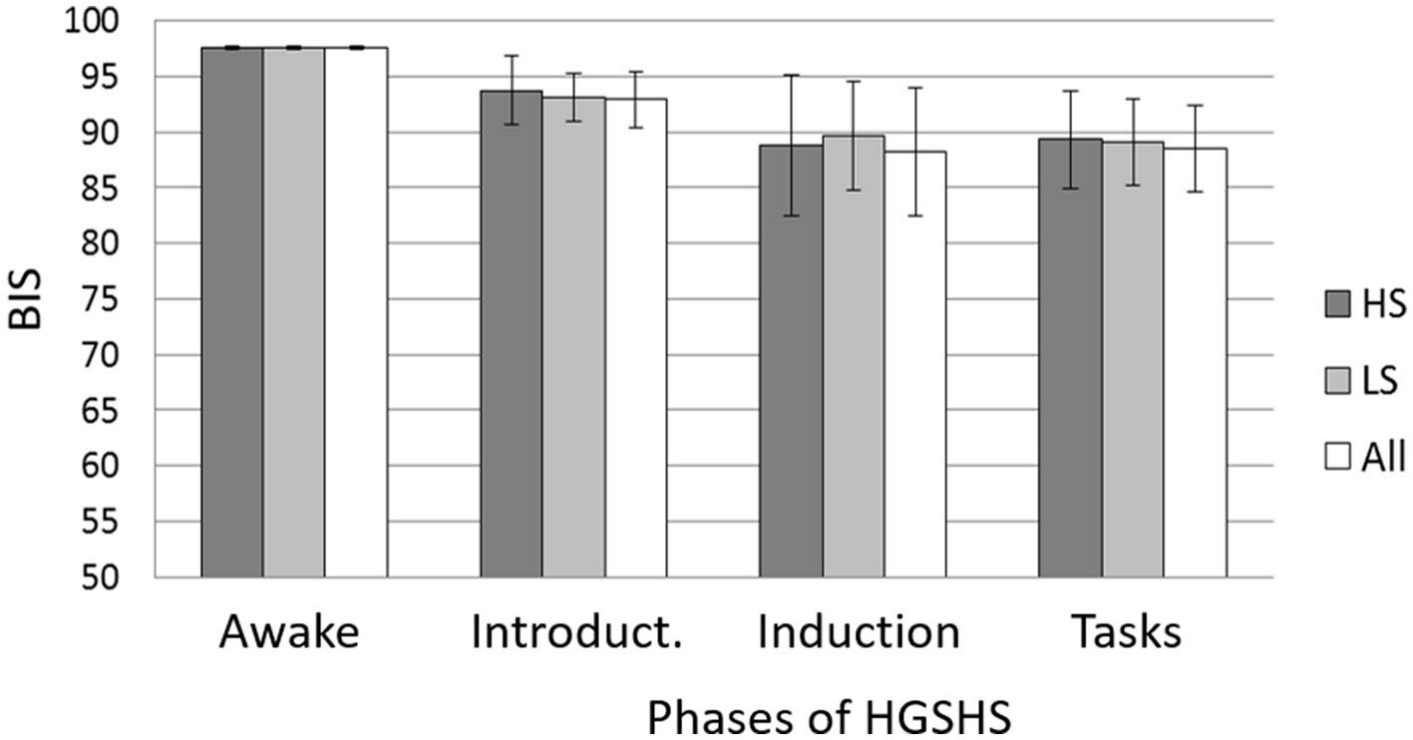
Mean BIS and SD during the different phases of the HGSHS testing compared between suggestibility groups. HS = high suggestibles (n = 15); LS = low suggestibles (*n* = 10); all (*n* = 56). Phases: awake, introduction (item 1), trance induction (item 2), and hypnotic tasks (items 3–11) (see Table 1). BIS = bispectral index score. Mixed ANOVA showed no statistical differences between the suggestibility groups.

Data were analyzed for different factors that may influence BIS and CSI values during HGSHS testing. Analyses were carried out with the three phases (A–C) described in the Methods section. There was no statistically significant influence of age group on BIS (z = 0.89, n.s.) or CSI (z = 1.13, n.s.) recording in any test phase. No statistically significant differences were found with respect to sex (BIS with z = 0.98, n.s. and CSI z = 1,20, n.s.). Only male participants showed a tendency to lower values in CSI recordings. There was no significant difference concerning the suggestibility group with regard to BIS (z = 0.03, n.s.) or CSI (z = 0.71, n.s.) values in the three phases. Figure 7 presents the comparison for BIS.

## 4 Discussion

### 4.1 Monitoring hypnotic depth

Hypnosis goes along with a very special subjective experience and a qualitative change in the consciousness of hypnotized individuals. Nevertheless, from the beginnings of modern hypnosis, attempts have been made to measure and quantify the hypnoidal state and hypnotic depth reached during the induction and maintenance of hypnosis (Perry and Laurence, 1980). Several methods have been developed for obtaining subjective depth estimates by instantaneous (LeCron, 1953) or retrospective self-rating (O'Connell, 1964; Pekala and Maurer, 2013). Other attempts were directed to objective measures of hypnotic depth based on visible behavior in response to suggestions given while people were hypnotized. Both measures became the basis for different scores of hypnotic susceptibility derived from self- or observer-ratings of a person‘s ability to respond to specific suggestions with sensorial or motoric reactions. In the search for more objective parameters to measure hypnotic depth as a state of consciousness and to monitor hypnosis during induction, maintenance, and interventions, a number of physiological reactions to hypnosis have been observed and evaluated. Some, like changes in skin conductivity or heart rate variability (De Benedittis et al., 1994; Kekecs et al., 2016), capture only specific, peripheral, limited effects. Functions of the central nervous system seem more promising to reflect hypnosis-induced effects on consciousness (Wolf et al., 2022). Neuroimaging revealed that the hypnotic state distinguishes from other (e.g., sleep) or non-ordinary (e.g., meditation and mindfulness) states of consciousness (Rainville et al., 2002; Vanhaudenhuyse et al., 2014). However, these techniques are rather elaborate and only of limited suitability for monitoring time courses. Moreover, more ideal for assessing general brain state changes such as wakefulness, sleep, and attentiveness are electrophysiological parameters (Jensen et al., 2015a). Trance-characteristic changes have been observed and described using frequency bands of electroencephalography (EEG) (Hinterberger et al., 2011; Jensen et al., 2015b) and event-related potentials of neuronal brain activities (Franz et al., 2020). Due to the multiple electrodes, calculations, and graphic representations, they are also rather complex and only limitedly suitable for monitoring, even less in medical or psychotherapeutic practice.

### 4.2 Monitoring of anesthetic depth

A similar challenge exists with the measurement of another non-ordinary state of consciousness, namely general anesthesia, i.e., pharmacologically induced hypnosis. There is a strong demand for monitoring of anesthetic depth, both to prevent traumatizing “intraoperative awareness” or unfavorable too-deep anesthesia and for the potential automatization of anesthesia (Stein and Glick, 2016; Roche and Mahon, 2021). Accordingly, several monitors based on EEG or evoked potentials have been developed. Feasibility by reducing multichannel EEG to a few electrodes and reducing EEG characteristics is gained at the expense of sensitivity and specificity for evaluating changes in brain activities. Although both goals, avoidance of awareness and anesthetic automatization, have not yet been achieved, monitors of narcotic depth are widely used in anesthesia and intensive care to get additional information and for training and education, with the most extensive distribution and research for BIS. The thereby derived indices are far from being specific. They also show changes in other states of consciousness, such as sleep and coma, or during relaxation, including acupuncture-induced tension release (Nieuwenhuijs et al., 2002; Fassoulaki et al., 2003). However, when such other causes are excluded, especially drug effects, and the interventions are limited to induction, maintenance, and deepening of hypnotic trance, then they could possibly serve to monitor non-pharmacological hypnosis as well.

### 4.3 Response of anesthetic depth scores to hypnosis

The results of this study show that BIS and CSI react to trance induction and maintenance of hypnosis (see Figure 3). This confirms an observation in 20 subjects of a drop in BIS index to an average of 87 after an unidentified trance induction (De Benedittis, 2008), later mentioned in an English review (De Benedittis, 2015). There also exist case reports from clinical applications of self-hypnosis or hypnotic communication (Burkle et al., 2005; Hansen et al., 2013) with drops in BIS down to a score of 75. In a diploma thesis, CSI monitoring showed significant decreases in the index after unspecified induction of relaxation or hypnotic trance, but no statistically significant difference between them (Bock, 2013). A subsequent pilot study reported that after an unidentified trance induction, significantly deeper CSI was observed in five highly suggestible subjects (starting from lower baseline values), whereas in four low suggestible subjects, CSI after relaxation or trance induction was raised even higher than the awake baseline (Haipt et al., 2017).

Of great significance is the fact that in the present study on 56 subjects, no random induction text was used, no random hypnotic interventions were used, and no random technique for deepening the trance was applied. Instead, we used a standardized method of hypnosis used worldwide, namely a test for hypnotic susceptibility. The text for the HGSHS has been translated into many languages, and norms have been evaluated for many different countries and groups (Bongartz, 1985; see Table 1 in Peter et al., 2015). HGSHS was chosen for this study because audio recordings are available that further standardize the test and for better comparability with other, preceding studies on monitoring trance inductions. Although HGSHS was originally developed for group testing, individual testing was used here for study feasibility and the subject's convenience. The equivalence of individual and group assessments has been shown (Bowers, 1993). During the test, a trance induction is followed by deepening suggestions and then by several tasks to be performed under hypnosis. This is exactly reflected in the courses of BIS and CSI (Figure 3): a continuous decrease in the indices followed by a slight increase and a rather constant level during the various sensorial and motoric tasks. This increase may be attributable to greater involvement of consciousness during hypnotic tasks, as well as a shift in the brain regions involved in the different motor and sensory tasks (De Benedittis, 2008).

### 4.4 Comparison of BIS and CSI

The values of CSI were consistently lower than those of BIS and showed more variation. This parallels the findings of studies on medical sedation or anesthesia with propofol, where a scale difference of 6–10 scale points, wider variability, and less reliability were observed for CSI than for BIS (Cortínez et al., 2007; Hoymork et al., 2007; Pilge et al., 2011; Herzog et al., 2021). The observed differences between BIS and CSI scores are in line with the fact that the two methods use different algorithms to extract EEG signals and transform them into scale values between 0 and 100. Both algorithms are kept more or less secret by the manufacturers, which makes comparison difficult. However, with the overlapping use of EEG parameters, the obtained results show substantial and significant correlation (Cho et al., 2018). This was confirmed in the present study, however, with a correlation coefficient of 0.38, equivalent to only a weak consistency (Figure 5). Moreover, the results verify the evaluation that the higher precision and reliability and more comprehensive scientific research of BIS outweigh the advantages in cost and portability of CSI.

### 4.5 Comparison to subjective hypnotic depth

In addition to the electrophysiological measurements, hypnotic depth was also evaluated by a retrospective subjective self-rating. The HD scores followed a course similar to the BIS and CSI monitoring (Figure 4) but with more variation in the level during task performances. This course during the test for HGSHS has been observed before (Perry and Laurence, 1980), with an increase of depth at task 4 and decreases at tasks 8 and 9 (see Table 1). These inconsistencies, in part, can be attributed to a connection between subjective scoring and task difficulty and experience. For instance, after failing to image a fly (task 9), participants could tend to rate their hypnotic depth low for that item. This substantially weakens the potential of hypnotic depth scoring toward a statement about “real” hypnotic depth. Such changes in depth values were also seen in the present study, in contrast to the course of BIS and CSI during the various items. Although HD and the electrophysiological measurements showed comparable overall courses during HGSHS testing (see Figures 3, 4) with regard to mean values, statistical analysis revealed no significant correlation. The reason for this seeming discrepancy lies in the fact that the corresponding test for linear regression, in contrast to the time course of mean values, compares the values of the individuals. Figure 5 shows exemplary BIS values and a selected phase (test items 2–5, with deep levels of both parameters suggesting effective hypnosis), demonstrating that while BIS and HD showed low average values compared to the awake baseline, subjects with low BIS values could have low and high HD values, and vice versa. In contrast to the time course of means that seem to “correlate,” the test for correlation between individual values of the two parameters was negative. On the other hand, the observed correlation between recorded trance depth and suggestibility scores confirms earlier reports (Perry and Laurence, 1980).

### 4.6 Influence of hypnotic suggestibility group

Besides its use as a standardized hypnotic intervention in this study, HGSHS testing results in a score of hypnotic susceptibility and accordingly in the division of participants into suggestibility groups, usually in high, medium, and low suggestibles. In the present study, the values of BIS and CSI during the various phases of HGSHS did not differ between high- and low-suggestible subjects (Figure 6). This is in line with a recent study that found that the power spectrum density of alpha, theta, and gamma bands does not support the relevance of the hypnotic induction to the highs' experience of hypnosis (Callara et al., 2023) and thus to hypnotic susceptibility scores. A definitive EEG-based signature for hypnosis and hypnotizability is not yet established (De Pascalis, 1999).

However, our results are in contrast to a report of a correlation between suggestibility and BIS response, with a mean BIS level of 85 in highs and 95 in lows (*p* < 0.1) (De Benedittis, 2008). A reason for this might be the use of the Stanford Scale of Hypnotic Susceptibility, Form C (SHSS:C) in the respective study. The two scales are not equivalent because their content of sensorial and motoric tasks shows only moderate correlation (Evans and Schmeidler, 1966; Register and Kihlstrom, 1986). Only 36% of subjects classified as highly suggestible according to the HGSHS reached the same group affiliation in the SHSS:C (Kihlstrom, 2015). Furthermore, it might be of critical importance whether the test subject is aware of his or her suggestibility group before the BIS monitoring or not (Callara et al., 2023). This holds for many studies where participants are pre-selected for high suggestibility, as is common in hypnosis research. The influence of expectation on hypnosis has been described and discussed (Kirsch, 2000; Pekala et al., 2010). In the present study, the monitored test only subsequently resulted in group assignments. While some authors have described no or low correlation between suggestibility test results and ratings of hypnotic depth (O'Connell, 1964), others have reported a significant correlation (Perry and Laurence, 1980).

### 4.7 Can depth of anesthesia monitors measure and monitor the depth of hypnotic trance?

Hypnosis can be seen as a state that enhances reactions to suggestions. Therefore, to measure and monitor hypnosis and accordingly for the distinction of various depths of hypnosis, both markers for this non-ordinary state of consciousness, whether neurophysiological or subjective, and the response to the suggestions are of interest. Originally, suggestibility tests were taken as measures of hypnotic depth (O'Connell, 1964). Meanwhile, they are understood to test the ability of a person to react to suggestions (Peter, 2023). However, the response to suggestions is dependent on both suggestibility and certain conditions that enhance this responsiveness. Such conditions are the hypnotic state (after hypnotic induction) that renders “suggestibility” to “hypnotizability,” or a “natural trance” induced by acute medical situations (Cheek, 1962). The performance after suggestions, namely the experience of hypnotic phenomena with regard to muscular, sensory, and cognitive functions, is therefore not strictly dependent on a formal hypnotic induction and hypnotic depth (Callara et al., 2023). Only in approximately 50% of cases is suggestibility enhanced by hypnotic trance induction (Kirsch, 2000). Moreover, in clinical situations, suggestibility scores affect hypnotic suggestions only to a limited extent (Barber, 1991; Montgomery et al., 2011). Similarly, in psychotherapy, neither actual hypnotic depth (state) nor general suggestibility (trait) seems to correlate well with the therapeutic results of hypnotic interventions, and striking hypnotherapeutic results and benefits are observed at light levels of hypnosis. The percentage of patients who benefit from hypnotic interventions in clinical settings far exceeds the percentage of individuals scoring in the high range of hypnotic suggestibility scales (Montgomery et al., 2011). Similar limitations are evident for using the subjective experience of hypnotic depth, as the definition of hypnotic depth is lacking. There are no criteria defined for the self-rating of hypnotic depth. What should it feel like? In summary, the interrelationships of suggestibility, hypnotizability, hypnotic responsiveness, hypnosis, and hypnotic depth are extremely complex and still under research and debate (Peter, 2023). Further clarification is necessary before the question of whether anesthesia depth monitors can reflect non-pharmacological hypnosis and hypnotic depth can be answered.

Even if many hypnotic phenomena have been shown to leave clear traces in neurophysiological measurements, such as activations in the visual cortex with visual hallucinations or an increase of theta waves in the EEG, neither neuroimaging with fMRT or PET nor electrophysiological monitoring allow us to describe the state of hypnosis with specificity (De Benedittis, 2015). There are neurophysiological correlates of hypnosis but no hypnosis-specific fingerprint. Rather, recent findings provide preliminary evidence regarding the variables that remain viable as factors that might facilitate hypnotic responses, that is, structural connectivity, hemisphere asymmetry, higher levels of theta bandwidth activity, expectancies, trait hypnotisability, motivation, absorptive capacity, rapport, and context (Jensen et al., 2015b). The role and the interactions of these variables, however, remain to be elucidated. Therefore, it is not surprising that if EEG changes are not hypnosis-specific, then EEG-derived scores such as BIS and CSI are not either.

For instance, slow-wave oscillations, mainly theta waves have been hypothesized to facilitate hypnotic responding, i.e., they would be associated with both hypnotic susceptibility (trait) and hypnotic reactions (state) (Jensen et al., 2015b). Other studies have found no differences between suggestibility groups (De Pascalis, 1999; Hiltunen et al., 2021). The picture is even more complex and contradictory with the other oscillation bands, such as alpha, gamma, or delta waves. Taken together, research findings concerning EEG correlates of hypnotisability, hypnotic induction, and hypnotic suggestions have been heterogeneous, inconsistent, and difficult to interpret (Halsband and Wolf, 2021). The reasons are manyfold, including the limitation of EEG to peripheral cortical brain processes because of the superficial location of the electrodes. Moreover, with regard to BIS and CSI monitoring, it has to be considered that they are restricted to frontal registrations.

We are not as convinced as other authors that the “BIS index can reliably measure and monitor the depth of hypnotic trance” (De Benedittis, 2015). However, we confirm that under controlled experimental conditions, when other effects on consciousness are excluded, they can reflect electrophysiological changes connected to induction, deepening, and maintenance of hypnosis, guide improvement of the involved techniques, and allow feasible online monitoring during hypnotic interventions.

## Data availability statement

The original contributions presented in the study are included in the article/supplementary material, further inquiries can be directed to the corresponding author.

## Ethics statement

The studies involving humans were approved by EC University of Regensburg, vote 13-101-0040. The studies were conducted in accordance with the local legislation and institutional requirements. The participants provided their written informed consent to participate in this study.

## Author contributions

NZ: Conceptualization, Formal analysis, Investigation, Methodology, Project administration, Supervision, Validation, Writing—original draft. MS: Conceptualization, Formal analysis, Writing—original draft. EH: Conceptualization, Formal analysis, Investigation, Methodology, Software, Supervision, Writing—original draft.
